# On the significance of Surfactant Protein-A within the human lungs

**DOI:** 10.1186/1746-1596-4-8

**Published:** 2009-03-12

**Authors:** Torsten Goldmann, Daniel Kähler, Holger Schultz, Mahdi Abdullah, Dagmar S Lang, Florian Stellmacher, Ekkehard Vollmer

**Affiliations:** 1 Division for Clinical and Experimental Pathology, Research Center Borstel, Borstel, Germany

## Abstract

Surfactant Protein-A (SP-A) is the most prominent among four proteins in the pulmonary surfactant-system. SP-A is expressed by alveolar epithelial cells type II as well as by a portion of non small cell lung carcinomas (NSCLC).

The expression of SP-A is complexly regulated on the transcriptional and the chromosomal level. SP-A is a major player in the pulmonary cytokine-network and moreover has been described to act in the pulmonary host defense.

By the use of cell culture or animal models the functional properties have been repeatedly shown in many aspects, often bearing surprising properties which strongly indicate the physiological importance of SP-A. To date SP-A is recognized as a molecule essential for pulmonary development, structure and function. An upcoming number of reports deals with the role of SP-A for pulmonary pathology. This article gives an overview about the state of knowledge on SP-A focused in applications for human pulmonary disorders and points out the importance for pathology-orientated research approaches using immunohistochemistry or *in situ *hybridization as promising methods to further elucidate the role of this molecule in adult lung diseases.

## Review

### Surfactant

The role of the surfactant system for the development of the human lung is known to be essential. Since it is synthesized by humans starting in the 28^th ^week of pregnancy and reaching functional levels in the 34^th ^week, surfactant-substitution-therapy is a fundamental part of the treatment of premature babies suffering from Infant Respiratory Distress Syndrome (IRDS)[1].

Pulmonary surfactant regulates dynamically the alveolar surface tension. The central role of the surfactant system for maintaining pulmonary function has been repeatedly shown by the use of cell culture or animal models [2].

Surfactant is a complex mixture of lipids, carbohydrates and four proteins (SP-A, SP-B, SP-C, SP-D). The initial descriptions of surfactant lead back to the 1950s, but little attention was given to the surfactant proteins until the 1980s [3]. The genes coding for these proteins are located on different chromosomes. SP-B and SP-C are similarly structured hydrophobic proteins participating in the adsorption of phospholipids at the alveolar border, which leads to rapid reduction of the surface tension. The hydrophilic proteins SP-A and SP-D are members of the collectins with C-type lectin domains. SP-D together with SP-A play a role in the pulmonary defense against Gram-negative bacteria [4].

### SP-A: biochemical properties and genetic organization

**Figure 1:**
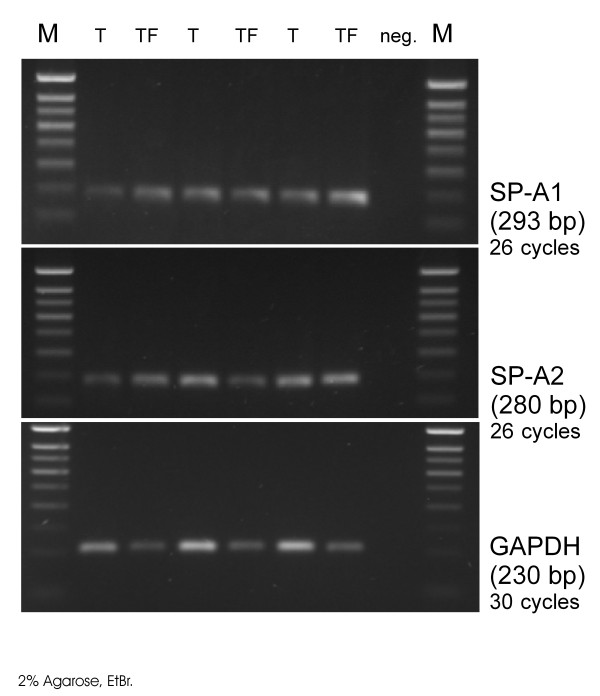
**RT-PCR: Transcription of SP-A1 and SP-A2 in NSCLC tumors (T) and corresponding tumor-free tissues (TF) from the same cases in comparison to GAPDH**.

SP-A is the major surfactant apoprotein exhibiting complex interactions and participation in processes fundamental for pulmonary structure and function with its expression restricted to alveolar epithelial cells type II. Moreover expression of SP-A was also described for a portion of NSCLC facilitating a diagnostic marker for these carcinomas [5,6]. After characterization of the biochemical properties, a complex chromosomal organization of the genes coding for SP-A has been demonstrated [3]. The locus of the SP-A on the one hand consists of two functionally active genes and a pseudogene [7,8]. The two active genes SP-A1 and SP-A2 on the other hand display several different alleles and splicing variants, moreover different oligomeric states have been described [3,9]. During the development of the lung these two genes are regulated differentially, a process triggered by cAMP and glucocorticoids [10]. The SP-A1 and SP-A2 genes display a homology of 94% in their nucleotide sequences and even 96% homology in the amino acid sequences [11]. Fig. 1, as one example, shows the transcriptional activity of the SP-A1 and SP-A2 genes determined by RT-PCR in homogenates of biopsies from NSCLC and corresponding tumor-free samples. The importance of the differential transcription of SP-A1 and SP-A2 for maintaining pulmonary function has repeatedly been demonstrated [12]. Phylogenetic analyses revealed that an ancestor proto-SP-A gene was diverged into SP-A1 and a second gene which subsequently emerged to SP-A2 and the SP-A pseudogene [3]. The high level of homology between SP-A1 and SP-A2 up to date prevents a differential analysis of the two gene products *in situ*. The expression of SP-A is also complexly regulated on the transcriptional level. Moreover the protein-turnover and the release of SP-A into the serum represents a further point of regulation [12]. This sophisticated regulation of the genetic activity is recognized as a further hint for the functional importance of SP-A.

### SP-A in pulmonary diseases

In recent years the role of defects in the expression of SP-A in context with different pulmonary diseases has become an issue of scientific investigations. Initially numerous studies have been performed to elucidate the role of surfactant substitution in pediatrics [2].

As one major function SP-A displays a protective role of the molecule in pulmonary host defense by interacting with various infectious agents such as bacteria, fungi and viruses. SP-A deficient knock-out mice – compared to wild type animals – are susceptible to infections with *Pseudomonas aeruginosa *[13] and the clearance of group B streptococcus is slower [14]. In accordance the defense of SP-A deficient mice against *Respiratory Syncytial Virus *(RSV) has been shown to be reduced and may be restored by exogenous SP-A administration [15-17].

By mediating the attachment of *Mycobacterium tuberculosis *to alveolar macrophages and promoting the phagocytosis of these bacteria, SP-A is important in the pathogenesis of tuberculosis [18-21]. SP-A also functionally interacts with staphylococci [22,23], *Haemophilus influenza Type A *[24,25], *Pneumocystis carinii *[26,27], *Influenza A Virus *(less efficient than SP-D) [28], *Candida tropicalis *[29] and *Aspergillus fumigatus *[30].

Moreover, SP-A is involved in the complex pulmonary network of cytokines as a central player, for example interacting with TNF-alpha and several interleukins [31,12,14].

Therefore it is likely that defects in the expression of SP-A may be important in the course of non infectious pulmonary diseases of adult patients. In the case of idiopathic pulmonary fibrosis, for example, low levels of SP-A (measured by ELISA) have been reported in broncho alveolar lavages (BAL), but elevated levels were found in the sera [32-34]. Immunohistochemical examinations of the expression of SP-A in pulmonary fibrosis demonstrated evident defects by using specimens from different diseases displaying fibrotic changes in the lungs. In good agreement with the results in BAL reduced levels of SP-A have been observed in fibrotic lungs. This reduced SP-A-expression in fibrotic lungs may be caused by two reasons: a limited number of the SP-A producing type II pneumocytes and by a clearly reduced SP-A expression of the remaining cells [35].

Reduced levels of SP-A have been demonstrated also in other pulmonary diseases such as the Adult Respiratory Distress Syndrome (ARDS) and in pneumonia [36].

Keeping in mind that surfactant substitutes are widely available due to their application in pediatrics, a growing number of therapeutic possibilities may result from these findings.

In sarcoidosis elevated levels of SP-A have been described [37] using BAL; the same applies for the sera from patients with alveolar proteinosis [38].

Since SP-A represents a central molecule in pulmonary immunoregulation as well as in host-defense it is obvious that defects in the surfactant system may have functional influence in the course of these pulmonary disorders.

**Figure 2:**
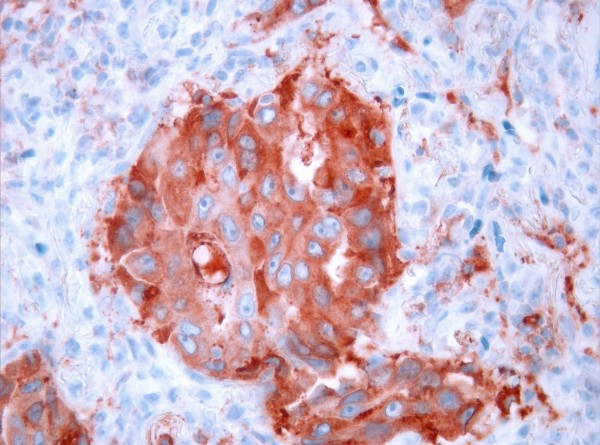
**Immunohistochemical detection of SP-A using the monoclonal antibody PE-10 (LSAB, amonoethylcarbazole, 400×)**.

Another point of research concerning SP-A is the diagnostic value of this molecule, the expression of which is restricted to the lungs. It has been reported that SP-A levels in BAL or serum from patients suffering from idiopathic pulmonary fibrosis correlate with the progression of the disease and can be used to predict survival [34,38]. In samples from airway secretions SP-A measurements are described to be useful also for the diagnostics of pulmonary edema where elevated levels have been found compared to healthy volunteers and ARDS patients [39]. By utilizing highly sensitive RT-PCR techniques the amplification of SP-A transcripts can be used for the detection of occult metastases in non small cell lung cancer patients [5,40]. Comparative studies of different malignomas with pulmonary localization have shown the diagnostic properties of immunohistochemically determined SP-A [6,41,42]. In carcinomas of occult origin localized in the lungs the diagnosis has a crucial influence on the therapy. A positive detection of SP-A represents a clear hint for a primary location in the lung [43]. Fig. 2 as an example shows the immunohistochemical detection of SP-A in a moderately differentiated adenocarcinoma of the lung using the primary monoclonal antibody PE-10 and LSAB (AEC-substrate, × 100). The positive staining in the tumor cells (reddish) in this certain case helped to manifest the diagnosis as a primary carcinoma of the lung.

However, the choice of a suitable SP-A antibody is highly important since approaches using polyclonals display cross reactions with other tumors [44]. This procedure has already become a part of pathological routine diagnosis, and – along with other markers such as the Thyroid-transcription-factor-1 (TTF-1) – the detection of SP-A (by PE-10) is a useful part of the immunohistochemical panel in pulmonary pathology.

Immunohistochemical detection of SP-A even might be utilized for forensic purposes helping to distinguish between fatal drowning and postmortem immersion [45].

## Conclusion

**Figure 3:**
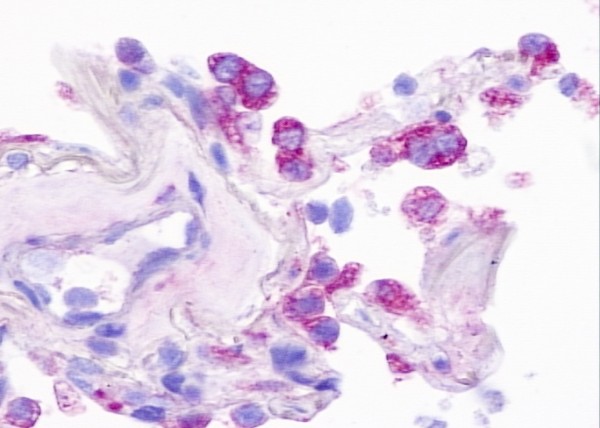
***In situ *hybridization targeting using a 663 bp digoxigenated DNA-probe complementary to SP-A mRNA**. Detection was achieved by Anti-digoxigenin antibody conjugated to alkaline phosphatase with NBT/BCIP as a chromogen (400×).

It is evident that SP-A is a molecule which already proves to be an interesting subject for medical research. However, the studies concerning the possible role of surfactant-defects in pulmonary diseases of adults have been performed mainly in different cell culture or animal models hardly analyzing adult human lung tissue. For these reasons SP-A is a promising target for histomorphological approaches using pathological specimens which exactly represent the scenarios of various diseases with all the different cell types involved which are difficult to simulate in models. With the modern tools of molecular pathology, the genetic activities of genes can be analyzed *in situ*, which provides evidence of the cellular activities in the context of a human native tissue. One example is shown in Fig. 3: a lung section hybridized with a digoxigenin-labeled SP-A probe to analyze the transcriptional activity *in situ*; the reddish signals of the transcripts are visible in the cytoplasm of type II pneumocytes. When analyzing the expression of SP-A in histological sections in context with other molecules of the pulmonary cytokine network one can expect further clues for the scenarios taking place in the course of interstitial lung diseases.

Taken together, SP-A is a complexly regulated molecule with surprising properties and essential importance for pulmonary development, structure and function which is getting more and more into focus concerning various diseases of the adult lung. Thus, as an outlook, it will become an issue of pulmonary pathology which might provide promising perspectives for applications in research, diagnosis and therapy.

## Competing interests

The authors declare that they have no competing interests.

## Authors' contributions

TG, DK and EV conceived the study. HS, MA, DSL and FS took part in the conception and writing of the manuscript. All authors read and approved the final manuscript.
